# Targeted IL-27-based gene therapy in preventing SARS-CoV-2 entry

**DOI:** 10.1007/s11033-026-12315-7

**Published:** 2026-07-11

**Authors:** Grace E. Mulia, Janelle W. Salameh, Marxa L. Figueiredo

**Affiliations:** 1 Department of Basic Medical Sciences, College of Veterinary Medicine, Purdue Institute for Drug Discovery, Purdue University, 625 Harrison St, LYNN 2177, West Lafayette, Indiana 47904 USA; 2 Purdue Interdisciplinary Life Sciences, Purdue University, West Lafayette, Indiana USA; 3 Weldon School of Biomedical Engineering, Purdue University, West Lafayette, Indiana USA

**Keywords:** SARS-CoV-2, COVID-19, Gene therapy, IL-27, ACE2-targeted therapy, Adipose-derived mesenchymal stromal cells

## Abstract

**Background:**

Coronaviruses such as SARS-CoV and SARS-CoV-2 have caused severe respiratory syndromes and prominent global health crises over the past two decades. Despite vaccines and antiviral therapies, treatment limitations persist, particularly in preventing viral entry and addressing emerging variants. ACE2, the primary receptor for SARS-CoV-2, represents a critical target for intervention. Cell-based approaches, including mesenchymal stromal cell therapies, have shown safety and promise in clinical trials. Building on our prior success with IL-27 gene therapy for acute respiratory distress syndrome, we explored ACE2-targeted IL-27 delivery as a potential strategy to reduce SARS-CoV-2 entry.

**Methods and Results:**

We developed an *in vitro* model using SARS-CoV-2 spike pseudotyped lentivirus to mimic viral entry. Human adipose-derived stromal cells were electroporated with plasmid DNA encoding either ACE2-targeted or non-targeted IL-27, and conditioned media were collected. Two regimens were tested: “prevention,” where cells were pre-treated with conditioned media before viral exposure, and “treatment,” where conditioned media and pseudotyped virus were added simultaneously. Viral entry was quantified using luciferase reporter activity and genome copy units in HEK293-ACE2 and A549-ACE2 cells. ACE2-targeted IL-27 showed a concentration-dependent trend toward reduced lentiviral entry, particularly in A549-ACE2 cells, although differences were not statistically significant.

**Conclusions:**

These findings provide proof-of-concept that ACE2-targeted IL-27 stromal cell-based gene therapy may help inhibit SARS-CoV-2 entry. Further optimization of dosing, delivery platforms, and testing in models that assess viral replication and inflammation, as well as evaluation against spike variants, are warranted to explore its potential as an adjunct to COVID-19 treatments.

**Supplementary Information:**

The online version contains supplementary material available at https://doi.org/10.1007/s11033-026-12315-7.

## Introduction

Severe acute respiratory syndrome (SARS) and related coronavirus infections have posed significant global health challenges over the past two decades, culminating in the COVID-19 pandemic caused by SARS-CoV-2. Coronaviruses are highly transmissible pathogens capable of inducing respiratory and systemic complications, with COVID-19 alone accounting for > 770 million confirmed cases and > 7 million deaths worldwide [1]. Despite the rapid development and deployment of preventative vaccines, effective antiviral therapies remain limited, emphasizing the need for host-directed strategies that can mitigate disease severity and improve outcomes. For instance, severe cases of SARS-CoV-2 infection are often associated with dysregulated immune responses, characterized by excessive cytokine production, which is referred to as a “cytokine storm.” Elevated levels of pro-inflammatory mediators such as IL-6, IL-8, IL-1, and TNF-α correlate with disease progression, multi-organ damage, and mortality [2]. Similar immunopathological features have been observed in other SARS-related infections, suggesting that therapeutic approaches aimed at restoring immune homeostasis may have broad applicability. As such, targeted immunomodulatory interventions capable of rebalancing cytokine profiles and reducing hyperinflammation may represent a promising avenue for combating severe coronavirus infections.

Interleukin-27 (IL-27) is a multifunctional cytokine that modulates numerous inflammatory signaling pathways. IL-27 has been shown to reduce inflammation in the context of allergic airway inflammation [3] and arthritis [4, 5] as well as prevent graft-versus-host disease [6]. In addition, IL-27 promotes the upregulation of anti-viral genes [7] and inhibits hepatitis B virus replication, helping to limit infection [8]. , IL-27 has been shown to modulate several interferon (IFN) pathways and interferon-stimulated genes (ISGs), helping to protect against Zika virus infection [9]. Furthermore, this cytokine is also capable of rebalancing both innate and adaptive immunity [10]; however, its therapeutic efficacy in the context of COVID-19 remains unexplored. Cytokines are promising, yet, utilizing them as therapeutics often requires repeated administration due to their short half-life [11]. Therefore, we propose a targeted gene delivery system using an IL-27 expressing plasmid. We chose a non-viral vector approach due to its cost-effectiveness, high reproducibility, and low risk of insertional mutagenesis to address potential biosafety concerns [12]. We recently demonstrated promising results using this approach to reduce inflammation in an LPS-induced ARDS mouse model [13]. To enhance the specificity of our therapeutic, we incorporated a novel, targeted form of IL-27 containing an ACE2-targeting peptide motif at the C-terminus. ACE2 is a receptor used by the SARS-CoV-2 virus to enter host cells and replicate [14]. We hypothesized that this targeted form of IL-27 could facilitate its delivery to cells infected by SARS-CoV-2, while also preventing viral entry and thereby reducing replication. To test this hypothesis, we determined whether the targeted form of IL-27 could reduce entry of a SARS-CoV-2 spike pseudotyped lentivirus in a cell culture model. This study provides proof-of-concept for ACE2-targeted IL-27 and adipose-derived stromal cells (ASCs) as a potential therapeutic for COVID-19.

## Materials and methods

### Cell culture

The human embryonic kidney cell lines expressing human angiotensin-converting enzyme 2 (HEK293T-hACE2) was obtained from BEI Resources (NR-52511). HEK293 and HEK293T-hACE2 cells were cultured in complete Dulbecco’s modified Eagle’s Medium (DMEM) (Corning, Corning, NY) supplemented with 10% Fetal Bovine Serum (FBS) (Hyclone, Cytiva, Marlborough, MA) and 1X Antibiotic-Antimycotic solution (Anti-Anti) (Gibco, Waltham, MA). HEK293T/17 cells were cultured in the DMEM (Corning), supplemented with 10% heat-inactivated FBS (ATCC), 2 mM L-glutamine (Corning), and 1X Anti-Anti on gelatin-coated plates. To apply the gelatin coat, 0.1% gelatin (StemCell Technologies, Vancouver, BC, Canada) was added to the tissue culture plates for 20 min, after which it was aspirated and air-dried under sterile conditions at room temperature (RT) for 2 h.

The human epithelial carcinoma cell line A549 and the mouse myoblast cell line C2C12 (from ATCC) were cultured in DMEM (Corning) supplemented with 10% FBS (Hyclone) and 1X Anti-Anti. Human lung carcinoma cells expressing human angiotensin-converting enzyme 2 (A549-ACE2) were from BEI Resources, NIAID, NIH (NR-53821). A549-ACE2 were grown in DMEM (Corning) supplemented with 10% FBS (ATCC), 1% MEM non-essential amino acid (Gibco), and 100 µg/mL Blasticidin (Gibco).

Human adipose mesenchymal stromal cells (hASC) from a male Caucasian donor (age 53, body mass index 30.02) were obtained from Obatala Sciences (New Orleans, LA). hASCs were cultured on fibronectin-coated plates in modified ASC medium, which consists of 60% DMEM (Corning), 40% MCDB-201 medium (Sigma-Aldrich, St. Louis, MO), 5% FBS (Hyclone), 1x insulin-transferrin-selenium (Corning), 1 nM dexamethasone (Thermo Fisher, Waltham, MA), 10 ng/mL epidermal growth factor (Gibco), 0.1 µM ascorbic acid (Thermo Fisher) and 1x Anti-Anti, following a previously outlined protocol [15].

### Vectors

To construct the vectors used in this study, pNFL1-secNluc (Promega, Madison, WI) was used as the backbone and linearized using Eco*RI*. The human IL-27elasti fragment from pUNO-97 hIL27(ebi3p28) (Invivogen, San Diego, CA) was cloned, with a 3’ insertion of a peptide linker sequence (G4S), followed by either the sequence for a non-ACE2 targeting peptide (pep7.1: DDVQAPNYTQHT) or an ACE2 targeting peptide (pep6.1: STSQKSIVAYTM) [16] using PCR cloning. The GFP expressing plasmid pDr5.GFP2 (Invivogen) was used as a reporter in several experiments through co-transfection. All vectors were purified using the GeneJET plasmid Maxiprep Kit (Thermo Scientific, Waltham, MA), according to the manufacturer’s protocols. To support the structural feasibility of our IL-27ACE2pep design, we performed *in silico* peptide-protein docking using the MDockPep2 server [17]. We utilized PDB entries 1R42 (human ACE2) and 6M0J (SARS-CoV-2 Spike/ACE2 complex) to simulate the binding configuration, yielding a highly favorable predicted interaction score (pepproscore) of -8.9. The resulting structures were visualized using UCSF Chimera to assess the interaction between the human ACE2 protein, SARS-CoV-2 spike protein, and the ACE2-targeting peptide (Fig. S1).

### *In vitro* transfection

C2C12 cells were seeded on 6-well plates at a density of 1.5 × 10^5^ cells/well and allowed to attach for 24 h. The next day, 2.5 µg of plasmid DNA was complexed with Lipofectamine 2000 (Invitrogen) at a 1:1 volume ratio and incubated for 20 min at room temperature. Following incubation, 500 µL of the DNA: lipid complex was added directly to the cells for 6 h. The media containing the complex was then replaced with complete growth media containing DMEM (Corning), 10% FBS (Hyclone), and 1X Anti-Anti (Gibco), and the cells were allowed to recover overnight.

For hASC transfection, a Neon transfection system (ThermoFisher) was used. A total of 5 µg of DNA, consisting of 4.5 µg of either an empty vector (pNLF1secnLuc), a non-ACE2 targeting IL-27 plasmid (pep7.1), or an ACE2-targeting IL-27 plasmid (pep6.1), complemented with 0.5 µg of a GFP-expressing vector (pDr5.GFP2), were used to transfect 4 × 10^5^ cells/well in a fibronectin pre-coated 6-well plate. Cells were co-transfected with the GFP-expressing plasmid, and successful transfection was verified using a fluorescent microscope the following day. Electroporation was performed with the following optimized settings: 1400 V, 10 ms pulse width, and 3 pulses. The day after transfection, the media was replaced with hASC complete growth media, and the cells were allowed to recover overnight.

### Cell Conditioned Media (CM)

To generate cell conditioned media, after the cell transfection and recovery period, the media was replaced with reduced-serum media consisting of Opti-MEM™ I Reduced Serum Medium (Gibco) and 1X Anti-Anti to promote IL-27 secretion from the transfected cells. After 24 h of incubation with reduced-serum media, this media was collected and referred to as conditioned media (CM). CM was collected every 24 h for up to 48 h and stored at -80 °C following centrifugation at 1500 rpm for 5 min to remove dead cells and debris.

### SARS-CoV-2 Spike protein pseudotyped lentivirus (SARS-CoV-2 LV particles)

Two SARS-CoV-2 Spike protein pseudotyped lentiviral particles, derived from the Wuhan-Hu-1 strain, were used in this study. The first was obtained from VectorBuilder (Fig S2). The second was generated in-house by adapting a protocol from Crawford *et al*. 2020 [18] using plasmids obtained from BEI Resources (NR-53816). The lentiviral particles were further concentrated using LentiX (Clontech, Mountain View, CA, USA), following the manufacturer’s protocol, and finally resuspended in 1X PBS (Corning) supplemented with 0.5% of Bovine Serum Albumin (BSA) (Jackson Immuno Research) at a volume of one-hundredth of the starting volume and stored at -80 °C.

The lentiviral titer was determined using flow cytometry. HEK293T-hACE2 cells were seeded at 5 × 10^4^ cells/well in a 24-well plate pre-coated with poly-L-lysine (Sigma-Aldrich). The cells were cultured in DMEM (Corning) supplemented with 10% heat-inactivated FBS (Hyclone), 2 mM L-glutamine (Corning), and 1X Anti-Anti (Gibco). To coat the plate, 300 µL of poly-L-lysine (Sigma-Aldrich) were added and then incubated for 5 min at RT, before being washed three times with sterile water and dried at RT for at least 2 h. About 24 h post-seeding, lentiviral particle dilutions were prepared at varying concentrations (0, 1:100, 1:1000, 1:10000) and complexed with polybrene (VectorBuilder) to a final concentration of 5 µg/mL. Cells were transduced with 300 µL of the diluted lentiviral particles for 6 h after which the media was replaced with complete growth media. Forty-eight to seventy-two hours post-transduction, wells with 1–10% positive cells were trypsinized and centrifuged at 300 × g for 4 minutes. The cells were washed twice with 3% BSA in PBS and resuspended in 1% BSA in PBS. The cells were then analyzed using flow cytometry (Attune NxT, Thermo Fisher). The titer was calculated using the Poisson Formula [18], i.e., titer per ml = -ln(1-P/100) * (number of cells/well)/ volume of virus per well in ml), where P = % of cells that are ZsGreen positive.

### SARS-CoV-2 LV entry assay

ACE2-expressing cells, either HEK293-ACE2 or A549-ACE2, were seeded in 96-well plates at a density of 1 × 10^4^ cells/well. Prior to seeding HEK293-ACE2 cells, the 96-well plate was pre-coated with 0.1% Gelatin (StemCell Technologies) for 20 min. After aspirating the solution, the plate was dried at RT for at least 2 h. The next day, depending on whether the experiment involved a “prevention” or “treatment” regimen, CM was added to the cells either 4 h prior to or simultaneously with the addition of SARS-CoV-2 lentiviral (LV) particles. Following the addition of LV particles, the plate was centrifuged at 1000 × g at 32 °C for 1 h to synchronize surface attachment by temporarily modulating endocytic kinetics [19]. Following spinoculation, cells were kept in an incubator at 37 °C and 5% CO_2_. Images (phase contrast and green fluorescence channels) were then captured using an IncuCyte live imaging system up to 48 h post-transduction.

To perform the trypsin cleavage and pH adjustments used in the A549-ACE2 LV entry assay experiments, the appropriate dilution of media with or without hASC CM, was first prepared, and the pH was adjusted accordingly using HCl and/or NaOH. Once the appropriate pH was reached, SARS-CoV-2 LV particles were added to the media and incubated with 1 µg/ml of TPCK-trypsin (ThermoFisher) for 30 min at 37 °C. Trypsin activity was then terminated by adding 10 µM of aprotinin (Sigma-Aldrich) as described in a previous report [20], before the mixture was added to the A549-ACE2 cells to begin the LV entry assay.

### Luminescence assay

After 48 h of transduction with SARS-CoV-2 LV particles, cells were lysed by adding 50 µL of 1X Passive Lysis Buffer (Promega, Madison, WI) and then freeze-thawed at -20 °C. In an opaque 96-well plate, 10 µL of cell lysate were added to 50 µL of luciferin substrate, and luminescence was measured using a GloMax plate reader (Promega) with a 10-second integration time.

### RNA extraction

To determine the effects of hASC IL-27 CM on gene expression, we conducted our experiment *in vitro* using ACE2-expressing cells, namely HEK293-ACE2 or A549-ACE2, and measured fold-change expression to assess a uniform, population-level response. First, the cells were seeded in 6-well plates at a density of 2 × 10 cells/well for HEK293-ACE2 or 1.5 × 10^5^ cells/well for A549-ACE2 cells. HEK293-ACE2 cells were seeded on gelatin-coated plates, as previously described. The next day, the media was removed, and cells were treated with one of the following for 48 h: control (growth media), CM control (OptiMEM + 1X AA), non-targeting IL-27 CM, or ACE2 targeting IL-27 CM. After 48 h, cells were trypsinized and centrifuged at 1500 rpm for 5 minutes, then the supernatant was aspirated to yield a ‘dry’ cell pellet. The RNA was extracted immediately, or from a cell pellet stored at -80 °C, using the Qiagen RNeasy kit (Qiagen, Germantown, MD) and eluted in 50 µL of Ultrapure DNase/RNase-free distilled water (Invitrogen).

### Reverse transcription quantitative polymerase chain reaction (RT-qPCR)

Following RNA extraction, reverse transcription was performed on 0.5 µg of RNA per sample using amfiRivert Platinum cDNA Synthesis Master Mix (GenDepot, Katy, TX). Once complementary DNA (cDNA) was obtained, RT-qPCR was performed by mixing 1 µL of cDNA with 2X KAPA SYBR Fast qPCR Master Mix (Roche, Indianapolis, IN) and 1 µM forward and reverse primers for the target genes. The primers used for RT-qPCR are listed in Table 1, with GAPDH serving as an endogenous control. The reaction was performed using the ViiA7 Real-Time PCR system (Thermo Fisher Scientific) with the following conditions: 95 °C for 3 min followed by 40 cycles of 95 °C for 3 s, 60 °C for 30 s, and 72 °C for 19 s. Data acquisition was performed using QuantStudio 3 software (Thermo Fisher Scientific).

**Table 1:** List of Primers used for RT-qPCR

Target gene	Sequence
Human GAPDH	Forward: 5’ -ACAACTTTGGTATCGTGGAAGG- 3’ Reverse: 5’ -GCCATCACGCCACAGTTTC- 3’
Human ACE2	Forward: 5’-ACAGTCCACACTTGCCCAAAT- 3’ Reverse: 5’ -TGAGAGCACTGAAGACCCATT- 3’
Human dACE2	Forward: 5’-GGAAGCAGGCTGGGACAAA- 3’ Reverse: 5’ -AGCTGTCAGGAAGTCGTCCATT- 3’
Human STAT1	Forward: 5’ -CAGCTTGACTCAAAATTCCTGGA- 3’ Reverse: 5’ -TGAAGATTACGCTTGCTTTTCCT- 3’
Human STAT3	Forward: 5’ -ACCAGCAGTATAGCCGCTTC- 3’ Reverse: 5’ -GCCACAATCCGGGCAATCT- 3’

### Statistical analyses

*In vitro* experiments were performed in 3–6 replicates per condition, and the data are represented as mean ± standard deviation (SD). Statistical analyses were performed using an unpaired t-test or ANOVA, with a p-value < 0.05 considered statistically significant.

## Results

### Impact of IL-27 CM of different origins on SARS-CoV-2 LV entry

**Fig. 1:**
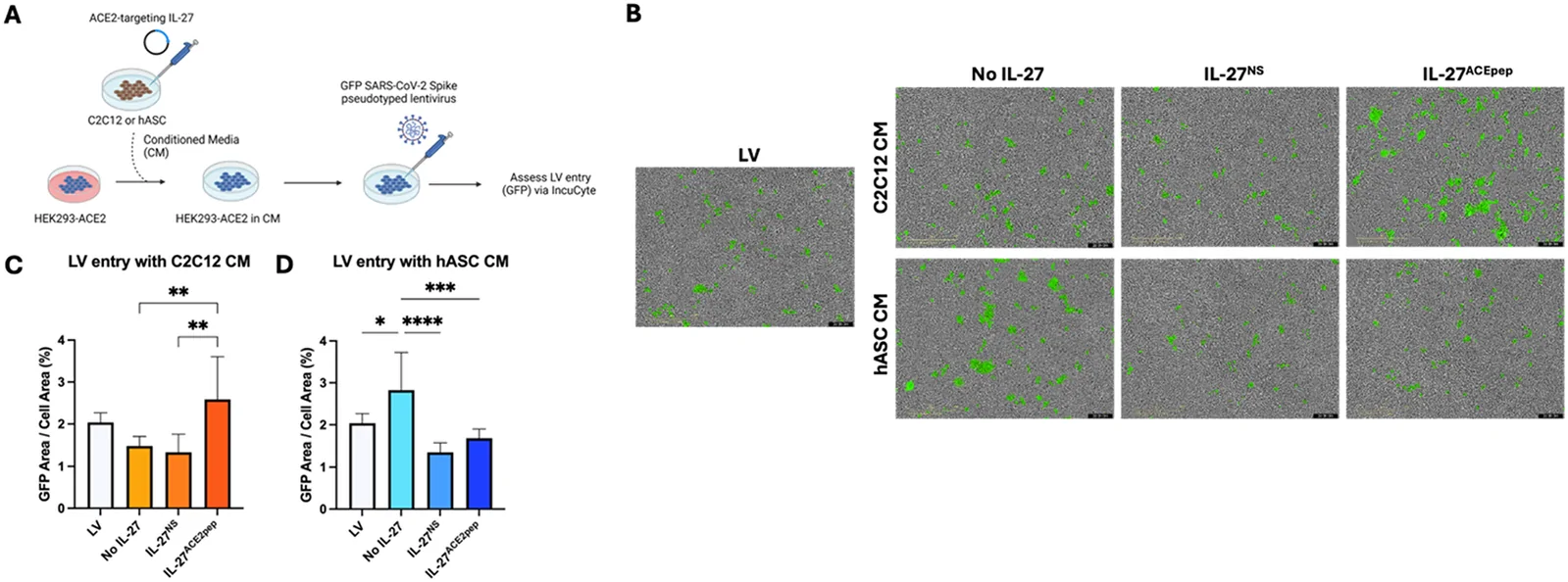
Effect of IL-27 conditioned media (CM) on SARS-CoV-2 pseudotyped lentivirus entry. (**A**) Schematic of the assay workflow (created with BioRender.com). SARS-CoV-2 Spike pseudotyped lentivirus (LV) expressing GFP was obtained from VectorBuilder. HEK293-ACE2 cells were treated for 4 h with growth media (LV), CM without IL-27 (no IL-27), CM with non-targeted IL-27 (IL-27^NS^), or CM with ACE2-targeted IL-27 (IL-27^ACE2pep^) prior to LV exposure. (**B**) Representative images of GFP expression at 48 h post-transduction indicating LV entry was measured using IncuCyte Live Cell imaging. CM was derived from C2C12 myoblasts (**C**) or human adipose stromal cells (hASC) (**D**). Data are mean ± SD; one-way ANOVA, *p < 0.05, **p < 0.01, ***p < 0.001, ****p < 0.0001

To evaluate whether IL-27 CM could reduce SARS-CoV-2 Spike pseudotyped LV entry, we tested LV entry in HEK293-ACE2 cells treated with IL-27 CM derived from either transfected skeletal myoblasts (muscle cells, C2C12) or human adipose mesenchymal stromal cells (hASC) (Fig. 1A). Our rationale for using these cell lines was based on their potential for clinical translation through intramuscular administration of gene therapy (C2C12 myoblasts as a model) or intravenous/intratracheal administration of gene therapy carriers (hASC as a model). Here, HEK293-ACE2 cells were treated with either growth media (LV) or conditioned media (CM) derived from C2C12 or hASC, which had been transfected with an empty vector (CM), a non-ACE2 targeting IL-27 plasmid (IL-27^NS^), or ACE2 targeting IL-27 plasmid (IL-27^ACE2pep^) for 4 h prior to the treatment with SARS-CoV-2 Spike pseudotyped lentivirus (SARS-CoV-2 LV). This SARS-CoV-2 LV was obtained commercially (VectorBuilder) and expressed green fluorescent protein (GFP). To assess LV entry, we monitored GFP expression using an IncuCyte Live Cell imaging system, with images taken every 4 h up to 48 h post transduction (Fig. 1B).

Interestingly, the trend in SARS-CoV-2 LV entry varied based on the origin of the CM. There was no significant difference observed between the control and C2C12 CM, but the C2C12 CM containing IL-27^ACE2pep^ unexpectedly promoted a significant increase in SARS-CoV-2 LV entry, (Fig. 1C). Meanwhile, hASC CM significantly increased SARS-CoV-2 LV entry into HEK293-ACE2 cells, and IL-27 expression in hASC CM significantly reduced SARS-CoV-2 LV entry, (Fig. 1D). Although there was no significant difference between the IL-27^NS^ and IL-27^ACE2pep^ hASC CMs on SARS-CoV-2 LV entry, these preliminary data suggested a potential role for IL-27 in modulating SARS-CoV-2 LV entry.

### Optimization of LV entry assay in HEK293-ACE2 cells

**Fig. 2:**
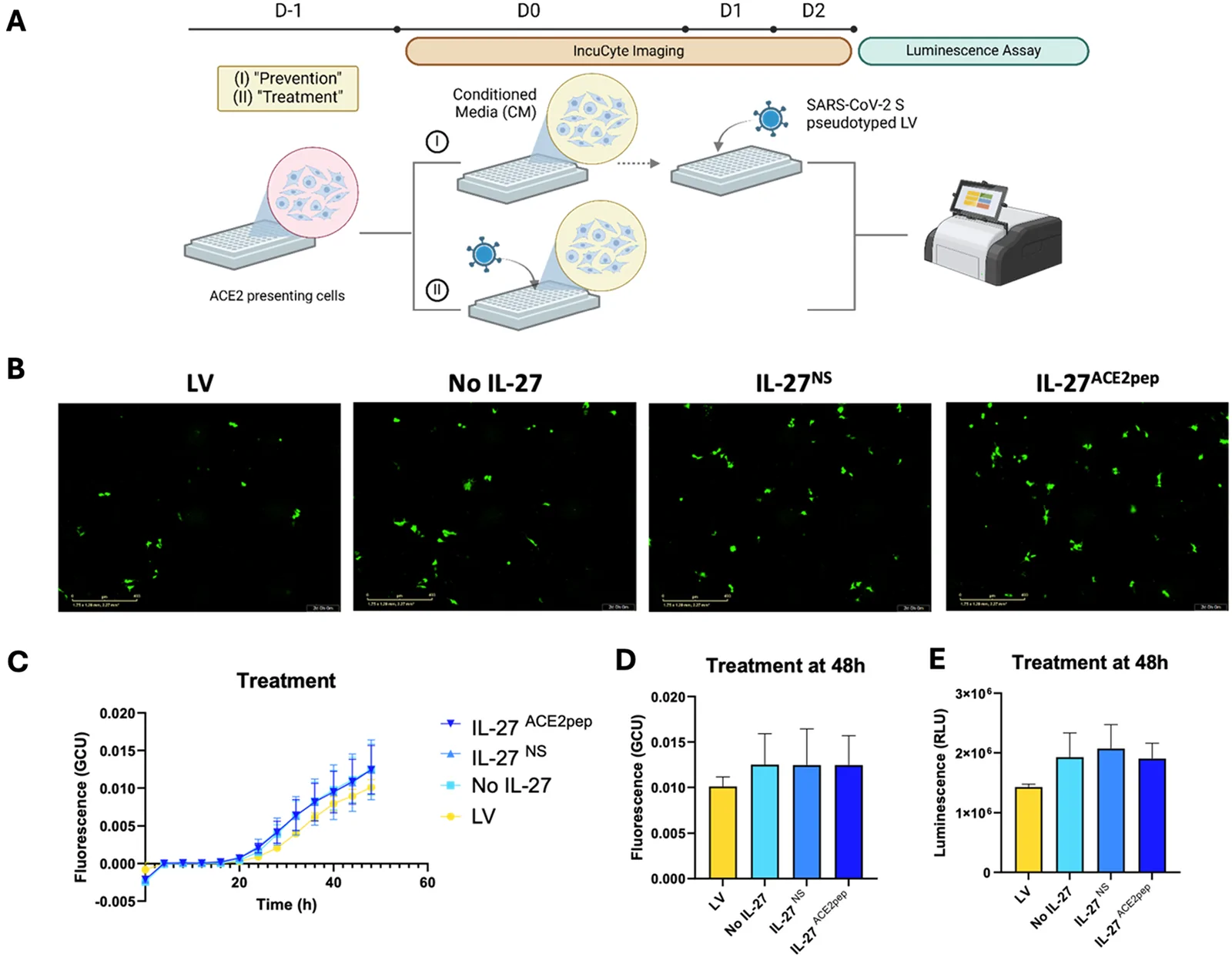
SARS-CoV-2 pseudotyped lentivirus entry in HEK293-ACE2 cells treated with IL-27 hASC conditioned media (CM) under a therapeutic regimen. (**A**) Schematic of the optimized assay with two regimens: prevention (CM added 4 h before LV) and treatment (CM added simultaneously with LV) (created with BioRender.com). Cells were imaged up to 48 h post-transduction using the IncuCyte system. (**B**) Representative images at 48 h for the treatment regimen. (**C–E**) LV entry quantified over time and at 48 h by GFP (**D**) and luminescence assay (**E**). Data are mean ± SD; one-way ANOVA, *p ≤ 0.05, **p ≤ 0.01, ***p ≤ 0.001, ****p < 0.0001

The initial LV entry assay showed limitations, with only ~ 1–3% GFP^+^ cells and high variability due to cell detachment, as reported by other groups [21]. To improve sensitivity, we replaced the commercial GFP-expressing SARS-CoV-2 pseudotyped LV (VectorBuilder) with an in-house LV generated using a BEI Resources kit (NR-53816). This LV includes a truncated spike to increase titers [18] and expresses ZsGreen1 and luciferase (Luc2), offering advantages: stronger fluorescence [22], endpoint luciferase verification, and higher sensitivity [23]. We also optimized HEK293-ACE2 attachment (Fig. S2) and introduced a “therapeutic” regimen, adding CM simultaneously with LV to mimic clinical relevance (Fig. 2A).

With the optimized model, LV entry increased over time across all treatment groups (Fig. 2C). No significant differences were observed between the control, no IL-27, non-ACE2-targeting, and ACE2-targeting IL-27 CM groups by IncuCyte imaging (Fig. 2B & D) or the luminescence assay (Fig. 2E). Luminescence results were comparable to GFP and offered greater sensitivity, so both methods were used in subsequent experiments.

### Effect of varying CM concentration on LV entry in HEK293-ACE2 cells

**Fig. 3:**
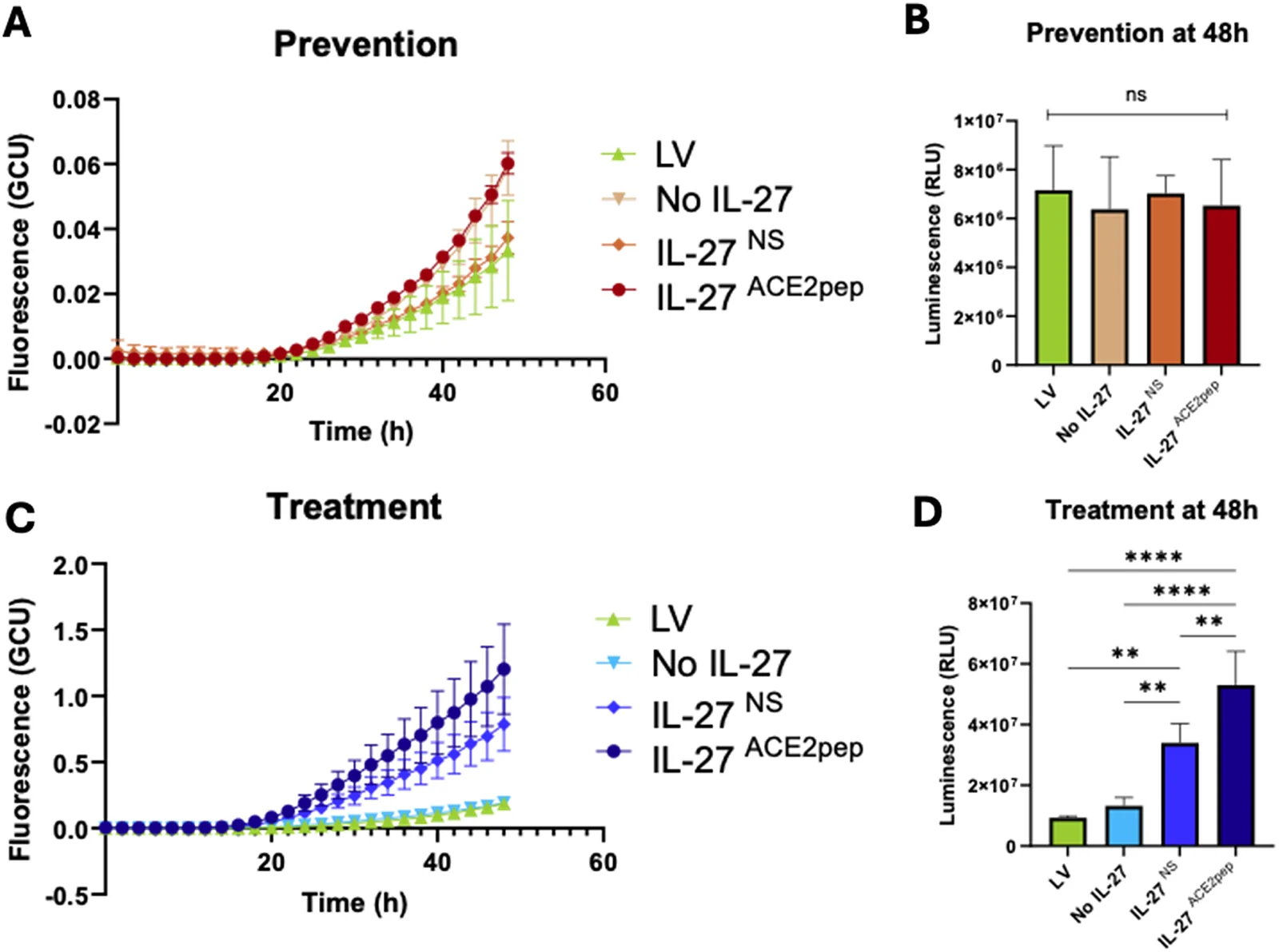
Effects of concentrated IL-27 hASC conditioned media (CM) on SARS-CoV-2 pseudotyped lentivirus entry into HEK293-ACE2 cells. Cells were treated with concentrated hASC CM (with or without IL-27) under prevention or therapeutic regimens. LV entry was assessed over 48 h by GFP intensity (Green Calibrated Units, GCU) using IncuCyte and at 48 h by luminescence (RLU). Data are mean ± SD; one-way ANOVA, *p < 0.05, **p < 0.01, ***p < 0.001, ****p < 0.0001

A 1:1 CM concentration previously showed no effect on LV entry. To test whether IL-27 levels or CM metabolites influence entry, we concentrated CM to 5× stock and evaluated LV entry under prevention and treatment regimens. In the prevention regimen, LV entry increased over time across groups, like the 1:1 condition (Fig. 3A). GFP readings indicated higher LV entry in the ACE2-targeting IL-27 and no IL-27 groups compared to the non-targeted IL-27 and LV controls, but luminescence data showed no significant differences (Fig. 3B). In the treatment regimen, LV entry was significantly higher with IL-27 CM compared to the LV control or no IL-27 CM (Fig. 3C-D), and ACE2-targeting IL-27 CM showed a significant increase compared to the non-targeted IL-27 CM.

**Fig. 4:**
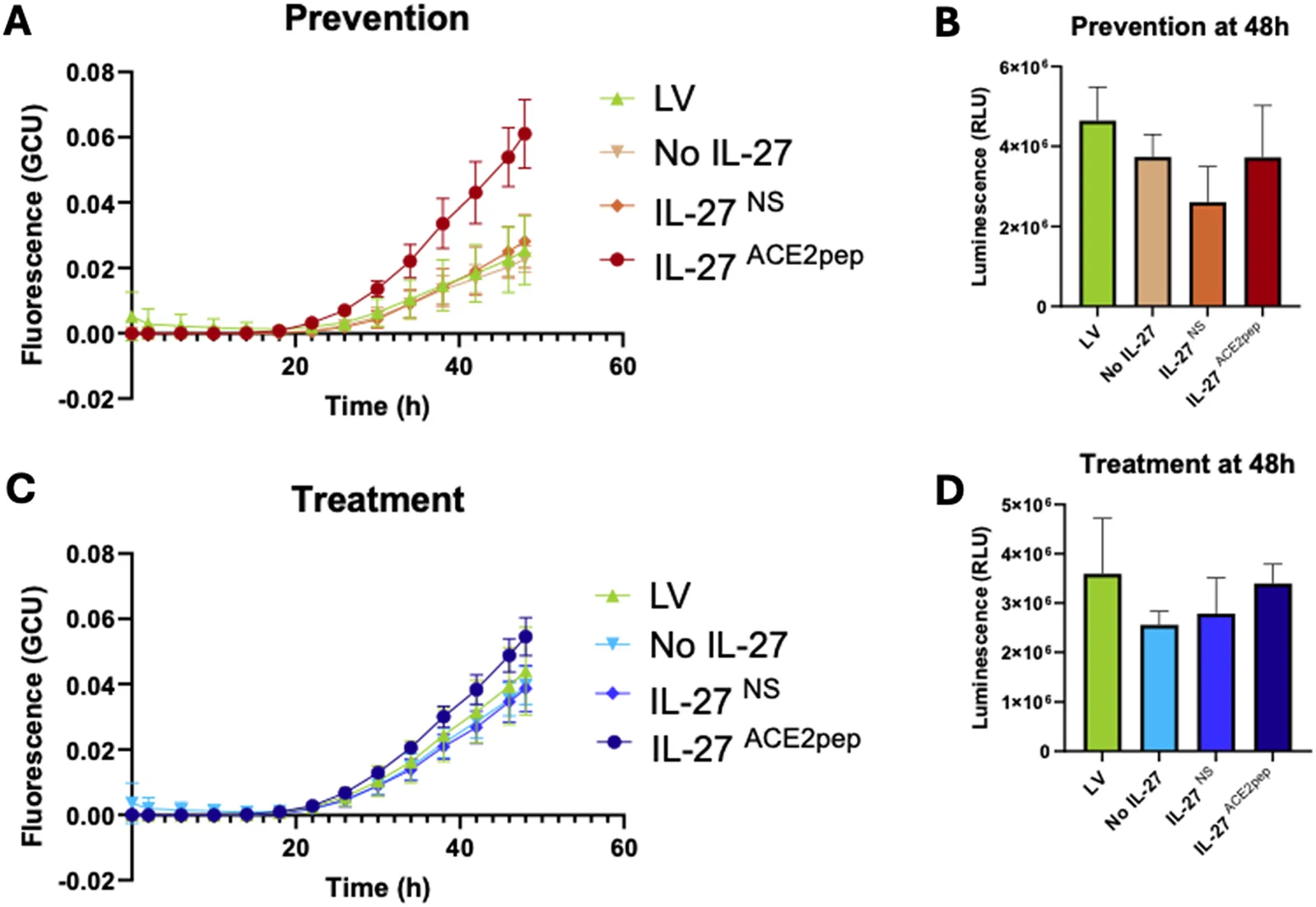
Effects of diluted IL-27 hASC conditioned media (CM) on SARS-CoV-2 pseudotyped lentivirus entry into HEK293-ACE2 cells. Cells were treated with diluted hASC CM (with or without IL-27) under prevention or therapeutic regimens. LV entry was assessed over 48 h by GFP intensity (Green Calibrated Units, GCU) using IncuCyte and at 48 h by luminescence (RLU). Data are mean ± SD; one-way ANOVA, *p < 0.05, **p < 0.01, ***p < 0.001, ****p < 0.0001

Next, we examined whether CM concentration correlated with LV entry by treating HEK293-ACE2 cells with diluted CM (1:10) under both prevention and therapeutic regimens. In the 1:10 condition, LV entry increased over 48 h post-transduction. Fluorescence data from both regimens showed that IL-27^ACE2pep^ groups had higher LV entry compared to LV control and IL-27^NS^ groups (Fig. 4A, C), though differences were not statistically significant. Luminescence assay results from the treatment regimen showed a similar trend (Fig. 4D). In contrast, the prevention regimen showed reduced LV entry with IL-27^ACE2pep^ and further decline with IL-27^NS^, but these changes were also not significant (Fig. 4B).

### IL-27 CM treatment induces STAT1-mediated ACE2 expression in HEK293-ACE2 cells

An increase in LV entry suggests that cellular changes may facilitate this process, including ACE2 expression levels. A previous study by Xiu *et al*. demonstrated that ACE2 expression is regulated through the STAT1 pathway [24], which also can be influenced by IL-27. Based on this, we hypothesized that IL-27 may affect ACE2 expression.

**Fig. 5:**
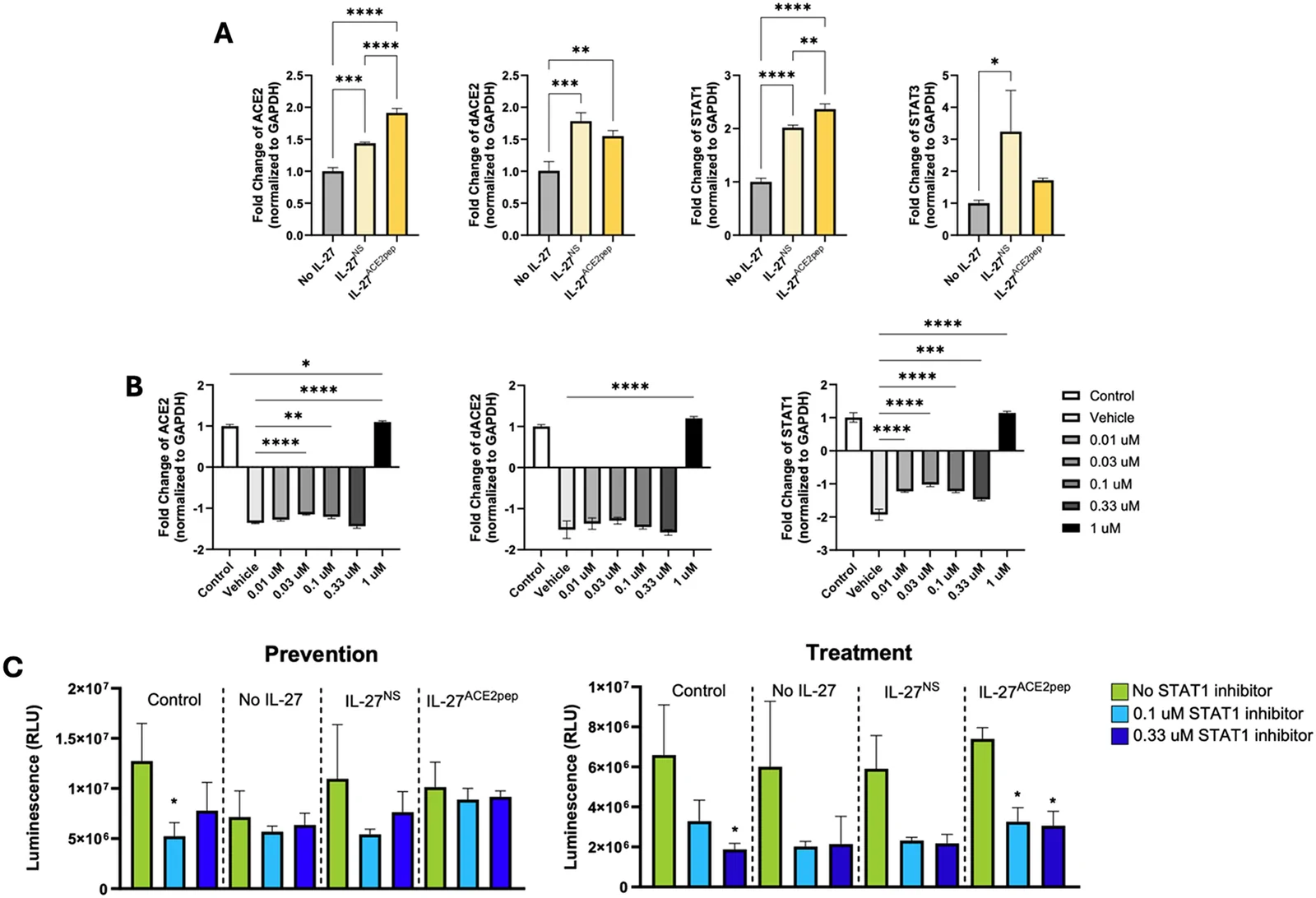
Effects of IL-27 CM and/or STAT1 inhibitor on gene expression and LV entry in HEK293-ACE2 cells. (**A**) HEK293-ACE2 cells were treated with or without IL-27 hASC CM (1:1) for 48 h. Gene expression was analyzed by RT-qPCR and normalized to GAPDH. (**B**) Cells were treated with PBS (control), DMSO (vehicle), or fludarabine (STAT1 inhibitor) at varying concentrations for 48 h prior to RT-qPCR analysis. (**C**) LV entry assay (prevention and treatment regimens) was performed in the presence of CM and/or STAT1 inhibitor; luminescence was measured at 48 h post-transduction. Data are mean ± SD (*n* = 3); one-way ANOVA, *p < 0.05, **p < 0.01, ***p < 0.001, ****p < 0.0001 relative to control or as indicated

To test this, we determined ACE2 expression in HEK293-ACE2 cells following treatment with various CMs using RT-qPCR. HEK293-ACE2 cells were treated with CMs containing no IL-27, IL-27^NS^, or IL-27^ACE2pep^ for 48 h prior to RT-qPCR analysis. We evaluated mRNA levels of ACE2, STAT1, STAT3, and dACE2, a truncated ACE2 isoform that does not impact viral entry [25]. Treatment with IL-27^ACE2pep^ CM resulted in a ~ 2-fold increase in ACE2 and STAT1 expression compared to the ‘no IL-27’ group (Fig. 5A). Meanwhile, dACE2 and STAT3 showed a ~ 1.5-fold increase following IL-27^ACE2pep^ treatment. These results suggested that IL-27^ACE2pep^ upregulates ACE2 expression in HEK293-ACE2 cells, potentially through the STAT1 pathway.

We then sought to determine if we could fine-tune ACE2 modulation by introducing a STAT1 inhibitor, fludarabine. Our hypothesis was that an optimized concentration of STAT1 inhibitor could reduce ACE2 expression while still allowing some IL-27-induced STAT1 signaling. We tested fludarabine at 0.01 µM, 0.03 µM, 0.1 µM, 0.33 µM, and 1 µM and measured ACE2, dACE2, and STAT1 mRNA levels. At the highest concentration (1 µM), fludarabine significantly upregulated ACE2, dACE2, and STAT1 expression (Fig. 5B). Lower concentrations (0.01–0.33 µM) also significantly upregulated STAT1 expression. Interestingly, 0.33 µM did not significantly upregulate ACE2 suggesting a potential concentration for modulating ACE2 while maintaining some STAT1 signaling. Thus, the results indicated that the relationship between STAT1 inhibitor and ACE2, dACE2, or STAT1 gene expression is not a simple dose-dependent response. We next repeated the LV entry assay with CM and fludarabine (0.1 and 0.33 µM). Both concentrations significantly reduced LV entry (Fig. 5C) but combining CM with fludarabine did not produce a synergistic effect. Microscopy revealed slower cell growth with fludarabine treatment, consistent with its known mechanism affecting the cell cycle [26]. Due to these growth effects, we could not conclusively establish a causal link between STAT1 inhibition and LV entry.

### Evaluating the effect of IL-27 CM on STAT1-mediated ACE2 expression in A549-ACE2 cells

**Fig. 6:**
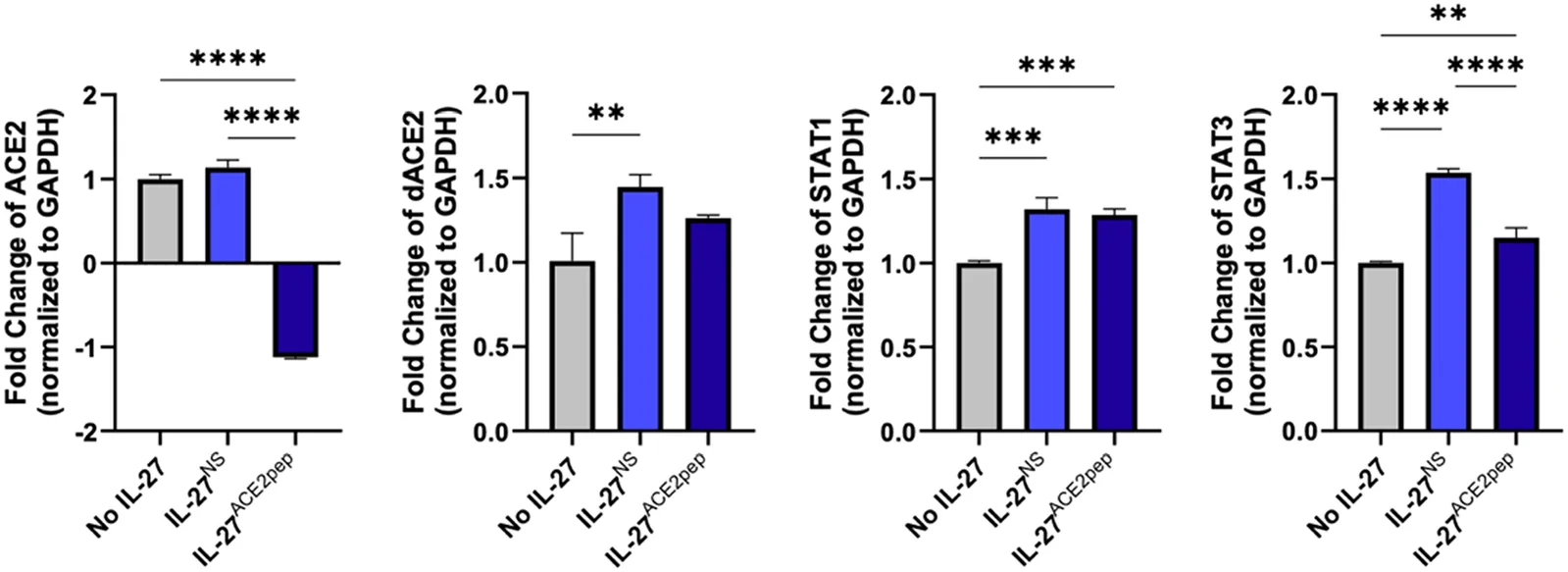
Effects of IL-27 hASC CM on gene expression in A549-ACE2 cells. A549-ACE2 cells were treated with IL-27 hASC CM (1:10) or control for 24 h. ACE2, dACE2, STAT1, and STAT3 expression was analyzed by RT-qPCR and normalized to GAPDH. Data are mean ± SD (*n* = 3); one-way ANOVA, *p < 0.05, **p < 0.01, ***p < 0.001, ****p < 0.0001

Next, we aimed to determine whether STAT1-mediated ACE2 expression occurs in other cell types, such as A549 cells overexpressing ACE2 receptors (A549-ACE2). We chose A549-ACE2 cells because they are derived from human alveolar epithelial cells, making them more relevant to the disease context. We treated A549-ACE2 cells with 1:10 diluted CM for 24 h and analyzed ACE2, dACE2, STAT1, and STAT3 expression by RT-qPCR. While IL-27 CM did not significantly alter ACE2 expression, it significantly upregulated dACE2, STAT1, and STAT3 (Fig. 6). Similarly, treatment with IL-27^ACE2pep^ CM significantly upregulated STAT1 and STAT3 expression, however, dACE2 showed only a slight increase, and ACE2 was downregulated. These results suggested that IL-27 CM may regulate ACE2 expression differently in A549-ACE2 relative to HEK293-ACE2 cells.

### Optimization of SARS-CoV-2 spike pseudotyped LV entry assay in A549-ACE2 cells

**Fig. 7:**
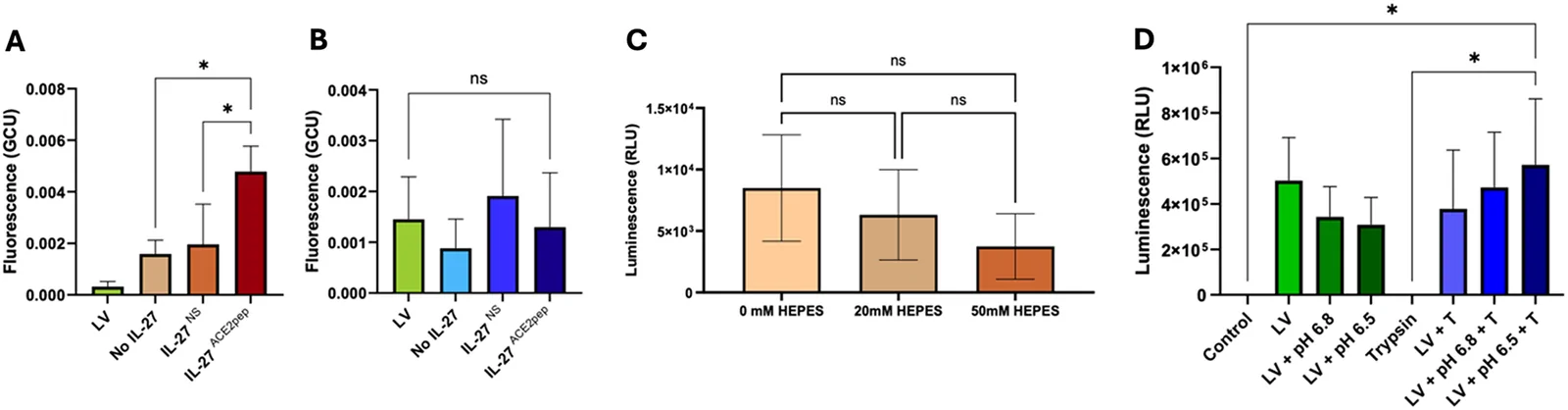
Optimization of SARS-CoV-2 pseudotyped lentivirus entry in A549-ACE2 cells. LV entry was assessed at 48 h under various conditions. (**A–B**) A549-ACE2 cells treated with SARS-CoV-2 Spike pseudotyped LV with or without CM (no IL-27, IL-27^NS^, or IL-27^ACE2pep^) in prevention (**A**) or therapeutic (**B**) regimens. (**C**) LV entry with varying HEPES concentrations. (**D**) LV entry at different pH values with or without trypsin cleavage of LV particles. Data are mean ± SD (*n* = 3); one-way ANOVA, *p < 0.05

Based on our previous findings regarding the potential use of A549-ACE2 cells in our model, we repeated our LV entry assay using the optimized conditions from the HEK293-ACE2 assays. Baseline fluorescence in A549-ACE2 cells was notably lower than in HEK293-ACE2 cells (Fig. 7A, B), approximately an order of magnitude lower (0.005 GCU vs. 0.010 GCU, respectively). This confirmed that SARS-CoV-2 entry requirements differ between cell lines [27, 28]. To further optimize LV entry in A549-ACE2 cells, we tested whether HEPES could improve entry, as reported by Chen S-H *et al*. [29]. Cells were treated with 0, 20, or 50 mM HEPES, but no significant differences were observed (Fig. 7C).

Other studies also noted that HEK293 cells require only the ACE2 receptor to allow for viral entry, while A549 cells require both ACE2 and TMPRSS2 [27, 28]. Kreutzberger *et al*. showed that entry improves at pH 6.8, which is clinically relevant (human intranasal pH 6.2–6.8), and that TMPRSS2 dependence can be bypassed by trypsin cleavage of LV particles [20]. We tested LV entry at pH 7.0, 6.8, and 6.5, with and without trypsin cleavage. pH alone did not significantly affect LV entry compared to pH 7.0 (Fig. 7D). However, combining pH 6.5 with trypsin cleavage significantly increased LV entry in A549-ACE2 cells. Based on these findings, we adopted this optimized condition for subsequent assays.

### Evaluating the effect of IL-27 CM on LV entry in A549-ACE2 cells

**Fig. 8:**
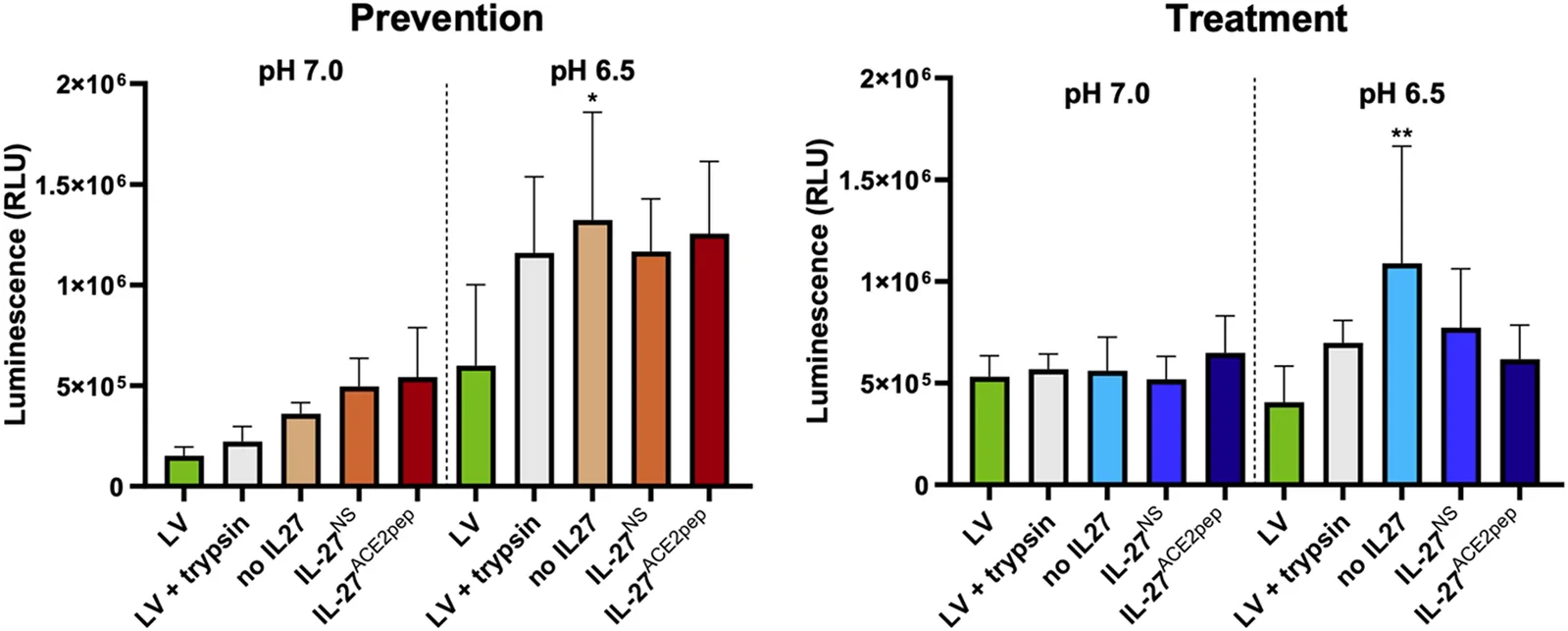
Effects of hASC CM with or without IL-27 on SARS-CoV-2 pseudotyped lentiviral entry in A549-ACE2 cells under optimized conditions. LV entry was assessed at pH 7.0 and pH 6.5 with trypsin cleavage under prevention and treatment regimens. Data are mean ± SD (*n* = 4); one-way ANOVA, *p < 0.05, **p < 0.01

We next evaluated the effect of IL-27 CM on LV entry in A549-ACE2 cells using the optimized conditions described above. Consistent with previous observations, both prevention and treatment regimens at pH 7.0 showed no significant differences between groups (Fig. 8). However, combining pH 6.5 with trypsin cleavage resulted in a higher baseline LV entry (~ 5 × 10⁵ RLU) in both regimens. Interestingly, hASC CM significantly increased LV entry in both regimens. The presence of IL-27 in hASC CM reduced LV entry, with a further reduction observed in IL-27^ACE2pep^ compared to IL-27^NS^, although this difference was not statistically significant. Overall, these results suggest that IL-27^ACE2pep^ hASC CM may warrant further exploration as a potential therapeutic approach for COVID-19.

## Discussion

Building upon the promising results from our previous work on using gene therapy for ARDS, this paper explores the therapeutic potential of a targeted form of IL-27 in the context of COVID-19. Specifically, we tested a novel IL-27 construct containing an ACE2-targeting peptide motif at the C-terminus to assess its ability to reduce SARS-CoV-2 viral entry. Essentially, we aimed to determine whether an ACE2-targeted IL-27 construct could inhibit viral entry *in vitro.* This study provides a first proof-of-concept for combining receptor-targeting with cytokine-based modulation in a SARS-CoV-2 entry model.

To investigate viral entry, we used a pseudotyped lentivirus, in which the Spike from coronavirus can be “pseudotyped” onto non-replicative viral particles, as reported in previous studies [30, 31]. However, the non-replicative nature of pseudotyped lentiviruses limits transduction efficiency compared to live virus models. Results from our study reported the baseline values of 0.010 GCU and 1.5 × 10^6^ RLU in HEK293-ACE2 cells, and 5 × 10^5^ RLU in A549-ACE2 cells. Similar pseudotyped-lentivirus protocols reported comparable or lower transduction efficiencies, ranging from 1.6 × 10^3^ to 1 × 10^5^ RLU in HEK293-ACE2 cells [32]. While several studies report data as percentage entry relative to control, we found that normalized RLU or GCU yielded similar statistical outcomes. Overall, our assay confirmed baseline transduction values consistent with prior reports, validating the model for subsequent testing. The pseudotyped lentivirus entry assay is generally used to evaluate the efficacy of neutralizing antibodies in blocking the Spike protein’s interaction with the host receptor (usually ACE2). Since our therapeutic involves an ACE2-targeting peptide motif, we modified the standard assay. We adapted an inhibition assay previously described [32], referred to as the “prevention” regimen, which allowed for the interaction of our therapeutic with the cells prior to the transduction of pseudotyped lentiviral particles. To improve the clinical relevance of our model, we also introduced a “treatment” regimen, in which the therapeutic and pseudotyped lentivirus were incubated simultaneously. This was designed to mimic a real-world scenario where therapy is given after infection, requiring competition with the virus. Thus, our modified assay allowed us to test both preventive and treatment scenarios, establishing a practical framework for evaluating antiviral biologics under pre- and post-exposure conditions.

Further optimization of our protocol also was specifically adjusted based on the cell lines used. We began with HEK293-ACE2 cells, which have been used widely for generating stable cell lines due to their high transduction efficiency [33]. However, these cells detach easily during washes, causing some assay variability; we overcame this by improving the cell attachment using gelatin-coated plates (Fig S3). In contrast, experiments with A549-ACE2 cells did not require such pre-coating, yet the presence of TMPRSS2 was more critical for regulating pseudotyped LV entry. We tested an alternative strategy to bypass TMPRSS2 activity using trypsin cleavage [20], which was successful. We further improved LV entry by lowering pH to mimic intranasal conditions (6.2–6.8), likely upregulating ACE2 as reported [34, 35]; whether these effects stem from ACE2 changes or other factors remains to be further characterized. These optimizations improved assay reliability and mimicked physiological conditions, though mechanistic details warrant further study.

Despite optimizing the LV entry assay in both HEK293-ACE2 and A549-ACE2 cells, we did not observe significant changes in LV entry following treatment of IL27^ACE2pep^ CM. In A549-ACE2 cells, we were able to detect a trend of reduced LV entry with the addition of IL-27 hASC CM, with a further trend of reduction observed in the presence of IL-27^ACE2pep^ hASC CM. Other studies have reported beneficial effects of using concentrated conditioned medium to reduce inflammation [36], which may align with some of our results. However, we observed that higher concentrations of IL-27 hASC CM did not consistently lead to the desired effect of reducing LV entry, as we had proposed. This supports findings from previous work where ACE2 expression was regulated by the activation of the STAT1 pathway [37], which IL-27 can modulate. Interestingly, we observed increased LV entry with IL-27^ACE2pep^, significant only at very high concentrations in the treatment regimen. This suggests that a certain threshold of IL-27 may be required to induce a significant effect on LV entry, and overall, IL-27^ACE2pep^ showed limited inhibition with unexpected concentration-dependent effects that warrant deeper dose-response investigation. Although effects were modest, these data identify dose and timing as key variables for refining IL-27-based antiviral strategies.

This then raises the question of whether the upregulation of ACE2 always correlates with worse outcomes in COVID-19 patients? Contrary to what we might expect, previous studies have shown that high expression of ACE2 is associated with increased viral entry, yet some studies suggest that high ACE2 levels could play a protective role in preventing severe acute lung injury or ARDS in COVID-19 patients [38, 39]. Additionally, coronaviruses have been shown to downregulate ACE2 expression, and also to induce ACE2 shedding [40], implying that this protein may have a broader protective role beyond serving simply as a receptor for viral entry.

Endogenous IL-27 may also play a greater immune-modulating role in COVID-19 patients, as IL-27 levels are typically upregulated, and low IL-27 levels have been correlated with poor prognosis [41]. IL-27’s antiviral properties include inducing IFN production in many immune cells and lung epithelial cells [42] by enhancing the transcription of ISGs, which are crucial in the context of SARS-CoV-2 infection, where type I IFN activity is dysregulated. IL-27 also can form complexes with IL-6 to modulate IFN expression [7], which may contribute to its protective effects. As our current model system used pseudotyped lentivirus particles and is replication-incompetent (as it cannot undergo viral replication and release), we can only focus on viral entry through this work. Thus, additional longitudinal ISG kinetic studies would be crucial to determine long-term immunomodulatory effects of our therapy. Taken together, ACE2 and IL-27 appear to have complex, context-dependent roles in COVID-19 pathophysiology, balancing viral entry and immune protection. This complexity supports exploring targeted immunomodulators like IL-27 as complementary strategies to antivirals, particularly for mitigating inflammation and tissue injury.

## Limitations

The scope of our *in vitro* experimental model focused on assessing a targeted form of IL-27 and its effect on viral entry using pseudotyped lentivirus particles. As our system is replication-incompetent, we acknowledge that we were unable to fully assess the therapeutic potential of the targeted form of IL-27 in COVID-19. Future studies could explore models assessing viral replication (e.g. using live virus) and inflammation (e.g., SARS-CoV-2–induced ARDS). Additionally, our gene therapy was tested using only one Spike variant (Wuhan-Hu-1 wild type). Since multiple variants with Spike mutations (D614G, N501Y, E484K, K417N/T) [43] have emerged, future work could evaluate IL-27 therapies against these variants.

The current results we obtained related to the STAT pathway also relied heavily on “transcriptional snapshot” of the pathway activation. This highlights the need to conduct future studies that can map the full temporal profile of STAT phosphorylation and translocation as well as characterizing downstream protein expression. Furthermore, longitudinal ISG kinetic studies would also be an important step in determining long-term immunomodulatory effects of our therapy.

Improvements can also be made to our plasmid design, such as using minicircle vectors, could enhance transgene expression and therapeutic efficacy compared to episomal plasmid DNA. Exploration of alternative targets like GRP78, which is upregulated during SARS-CoV-2 infection [44], may offer additional benefits, though IL-27’s impact on GRP78 requires investigation [45]. In combination, these insights lay the groundwork for next-generation strategies, refining dose and delivery, expanding to replication and inflammation models, testing across variants, and optimizing vectors to more fully define the therapeutic potential of targeted IL-27 stromal cell-based gene therapies.

## Conclusion

In the context of COVID-19, our targeted IL-27 stromal cell-based gene therapy demonstrated a trend toward reducing SARS-CoV-2 Spike pseudotyped LV entry *in vitro*, highlighting its potential as an antiviral strategy. Significant effects occurred only with high, sustained IL-27^ACE2pep^ CM concentrations (48 h), suggesting a possible threshold requirement. These findings emphasized the need for dose optimization and delivery strategies that can maximize therapeutic benefit. While limited to two cell lines and a single Spike variant, these trends provide a foundation for future work. Expanding to replication and inflammation models, variant-specific responses, and improved vector designs could unlock the full therapeutic potential of targeted IL-27 stromal cell-based gene therapies in combating COVID-19 and related viral diseases.

## Supplementary Information

Below is the link to the electronic supplementary material.


Supplementary Material 1



Supplementary Material 2


## Data Availability

Data is available upon reasonable request from the corresponding author.

## Abbreviations

ACE: Angiotensin converting enzyme
ACE2: Angiotensin converting enzyme 2
Anti-Anti: Antibiotic-antimycotic
ARDS: Acute Respiratory Distress Syndrome
ASC: Adipose derived mesenchymal stromal cell
ATCC: American Type Culture Collection
COVID-19: Coronavirus disease of 2019
DMEM: Dulbecco’s modified Eagle’s Medium
EBI3: Epstein-Barr virus induced gene 3
FBS: Fetal Bovine Serum
hASC: Human adipose-derived stromal/stem cells
IFN: Interferon
IL: Interleukin
ISGs: Interferon stimulated genes
LPS: Lipopolysaccharide
RT: Room temperature
S: Spike
SARS-CoV: Severe acute respiratory syndrome related coronavirus
SARS-CoV-2: Severe acute respiratory syndrome related coronavirus 2
STAT: Signal transducer and activator of transcription
TMPRSS2: Transmembrane protease serine 2
TNF: Tumor necrosis factor
WHO: World Health Organization
