# Correction: Targeted IL-27-based gene therapy in preventing SARS-CoV-2 entry

**DOI:** 10.1007/s11033-026-12683-0

**Published:** 2026-08-29

**Authors:** Grace E. Mulia, Janelle W. Salameh, Marxa L. Figueiredo

**Affiliations:** 1 Department of Basic Medical Sciences, College of Veterinary Medicine, Purdue Institute for Drug Discovery, Purdue University, 625 Harrison St, LYNN 2177, West Lafayette, IN 47904 USA; 2 Purdue Interdisciplinary Life Sciences, Purdue University, West Lafayette, IN USA; 3 Weldon School of Biomedical Engineering, Purdue University, West Lafayette, IN USA

**Correction: Molecular Biology Reports (2026) 53:1154**

**https://doi.org/10.1007/s11033-026-12315-7**

In this article, the author’s name Janelle W. Salameh was incorrectly written as Janelle E. Salameh. 

The original article has been corrected.

