# Supplementary material for: Targeted IL-27-based gene therapy in preventing SARS-CoV-2 entry
Source: Mol Biol Rep. 2026 Jul 11;53(1):1154. doi: 10.1007/s11033-026-12315-7 (PMC13356059; doi:10.1007/s11033-026-12315-7)
Supplement: Supplementary file 1 — Supplementary Material 1 [file 11033_2026_12315_MOESM1_ESM.docx]

**Supplementary Figures**


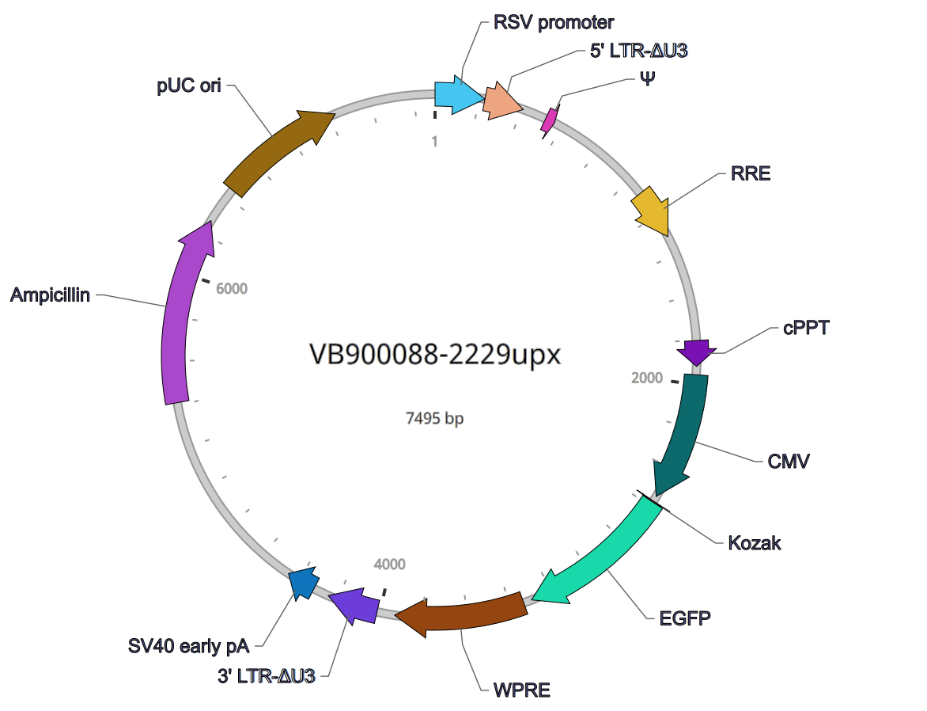


**Fig S1. SARS-CoV-2 Spike protein pseudotyped lentivirus** obtained from VectorBuilder.


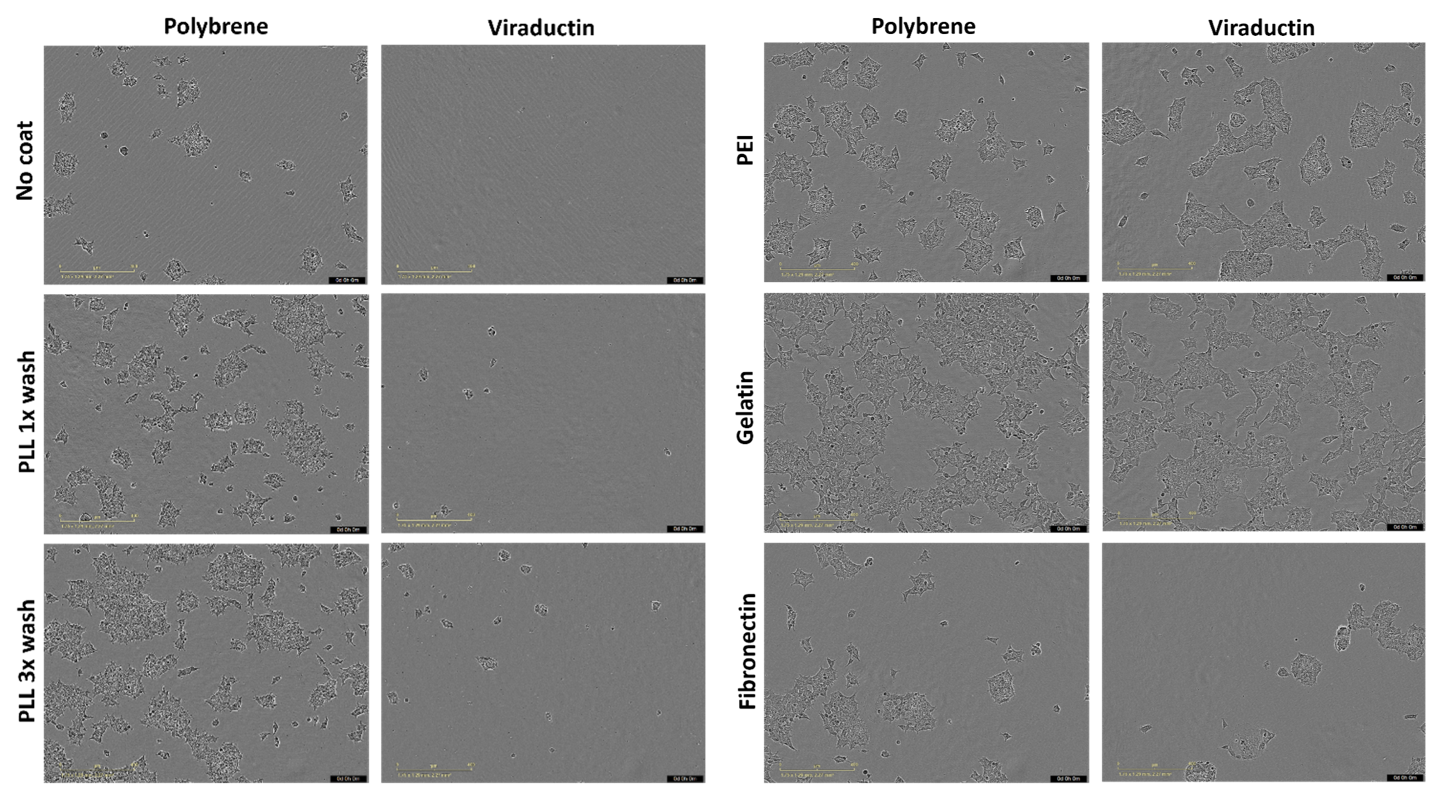


**Fig S2.** **Optimization of HEK293-ACE2 attachment conditions.** HEK293-ACE2 cells (1 x 10^5^ cells/well) were seeded in a 96-well plate that was either not pre-coated, pre-coated with poly-L-lysine (PLL) and washed once with distilled water (PLL 1x wash), PLL washed three times with distilled water (PLL 3x wash), polyethlenimine (PEI), gelatin, or fibronectin. After 24 h, cells were treated with either polybrene or viraductin to mimic the addition of transduction reagents during the LV entry assay. Images were taken using the IncuCyte live imaging system.
