# Supplementary material for: Targeted IL-27-based gene therapy in preventing SARS-CoV-2 entry
Source: Mol Biol Rep. 2026 Jul 11;53(1):1154. doi: 10.1007/s11033-026-12315-7 (PMC13356059; doi:10.1007/s11033-026-12315-7)
Supplement: Supplementary file 2 — Supplementary Material 2 [file 11033_2026_12315_MOESM2_ESM.docx]

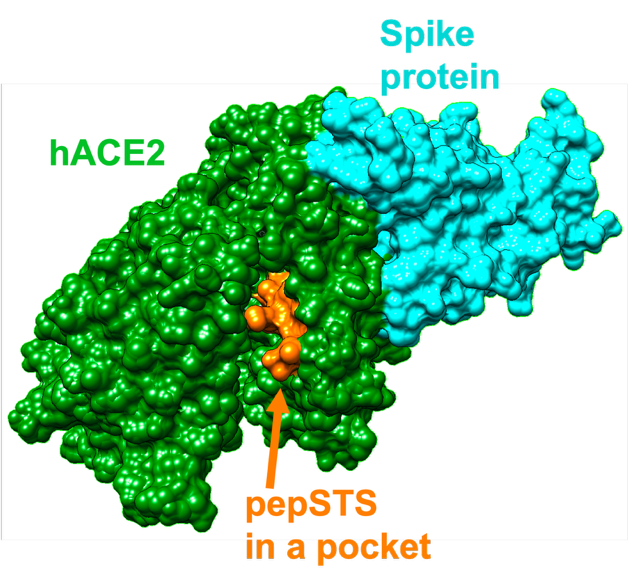
**Supplementary Materials**

**Fig S1*. In silico* peptide-protein docking of the IL-27ACE2pep design.** Docking was performed via MDockPep2 using PDB entries 1R42 and 6M0J. The predicted binding configuration yielded a favorable pepproscore of -8.9, indicating stable interaction between the human ACE2 protein, SARS-CoV-2 spike protein, and the targeting peptide. Structures were visualized using UCSF Chimera


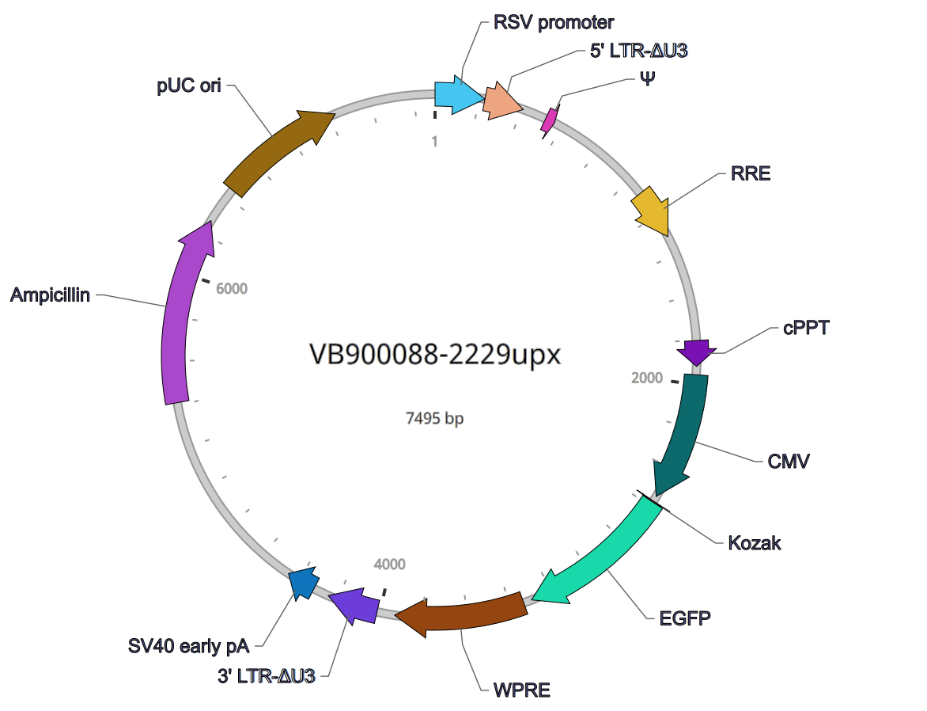


**Fig S2. SARS-CoV-2 Spike protein pseudotyped lentivirus** obtained from VectorBuilder.


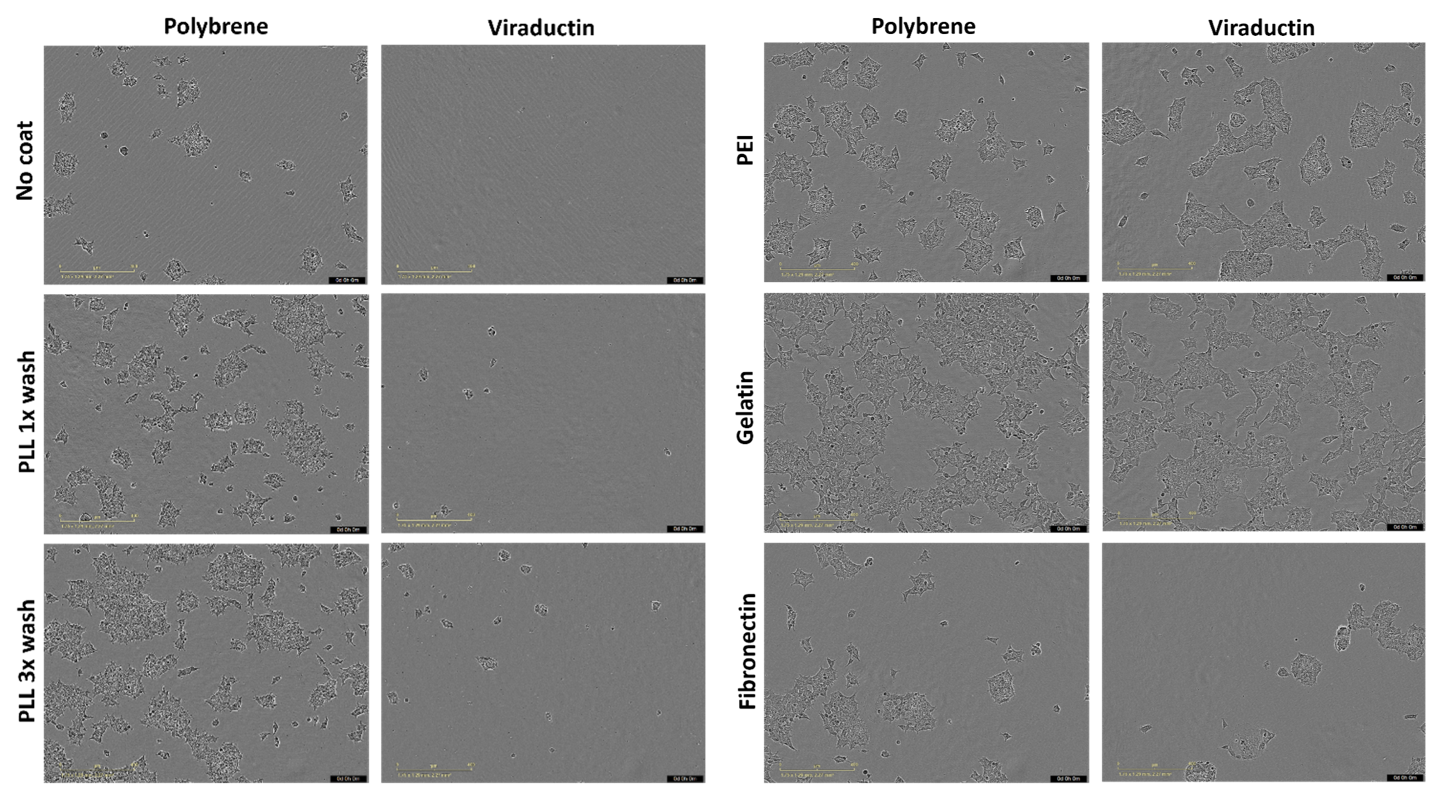


**Fig S3.** **Optimization of HEK293-ACE2 attachment conditions.** HEK293-ACE2 cells (1 x 10^5^ cells/well) were seeded in a 96-well plate that was either not pre-coated, pre-coated with poly-L-lysine (PLL) and washed once with distilled water (PLL 1x wash), PLL washed three times with distilled water (PLL 3x wash), polyethlenimine (PEI), gelatin, or fibronectin. After 24 h, cells were treated with either polybrene or viraductin to mimic the addition of transduction reagents during the LV entry assay. Images were taken using the IncuCyte live imaging system.
